# Supplementary material for: Architecture and self-assembly of the jumbo bacteriophage nuclear shell
Source: Nature. 2022 Aug 3;608(7922):429–35. doi: 10.1038/s41586-022-05013-4 (PMC9365700; doi:10.1038/s41586-022-05013-4)
Supplement: Supplementary file 1 — This file contains Supplementary Fig. 1 and Tables 1–10. The Supplementary Figure shows an alignment for the 201phi2-1 and PhiKZ chimallin. Table contents: cryo-EM map statistics, coordinate model statistics, protein–protein interface statistics, hinge residues, pore-facing residues, sequences used to construct the tree in Fig. 5a, and the bacterial strains and plasmids used in this study. [file 41586_2022_5013_MOESM1_ESM.pdf]

nature portfolio

https://doi.org/10.1038/s41586-022-05013-4

Supplementary information

Architecture and self-assembly of the jumbo bacteriophage nuclear shell

In the format provided by the authors and unedited

Supplementary Figure 1. Alignment of 201phi2-1 and phiKZ chimallin proteins. Alignment of 201phi2-1 gp105 and phiKZ gp54 protein sequences. Secondary structure and domain annotation are shown above the alignment and are based on the 201phi2-1 structure. Dashed lines in the domain annotation indicate unresolved segments. The phiKZ prohead protease (gp175) cleavage-site in gp54 is indicated by a yellow arrow. Cleavage-site based on that identified by Weintraub *et al.*[20].

SI Table 1. *In situ* cryoFIB-ET data collection and reconstruction statistics.

SI Table 2. Cryo-EM data collection, reconstruction, and refinement statistics. Particle counts for C4 and C1 refinements are for the total number of sub-particles.

SI Table 3. Summary of *in vitro* 201phi2-1 chimallin protomer interfaces. Chain 'A' corresponds to the position of the focal yellow colored chain and all other chains match the color scheme in **Extended Data Figure 5.**

SI Table 4. Summary of a 201phi2-1 chimallin protomer's polar interactions *in vitro.* Chain 'A' corresponds to the position of the focal yellow colored chain and all other chains match the color scheme in **Extended Data Figure 5.**

SI Table 5. Gaussian network model hinge residues for 201phi2-1 chimallin.

SI Table 6. Pore-facing residues during 201phi2-1 chimallin simulations. Pore-facing residues are defined as residues exposing their amino acid side chains to the channel pore more than 50% over the course of the averaged 300-ns simulations.

SI Table 7. Bacteriophage chimallin proteins. Asterisks (*) denote sequences included in the phylogenetic tree in **Figure 5a**.

SI Table 8. Summary of *in vitro* Goslar chimallin protomer interfaces. Chain 'A' corresponds to the position of the focal yellow colored chain and all other chains match the color scheme in **Extended Data Figure 5.**

SI Table 9. Summary of a Goslar chimallin protomer's polar interactions *in vitro.* Chain 'A' corresponds to the position of the focal yellow colored chain and all other chains match the color scheme in **Extended Data Figure 5.**

SI Table 10. Bacterial strains and plasmids used in this study.

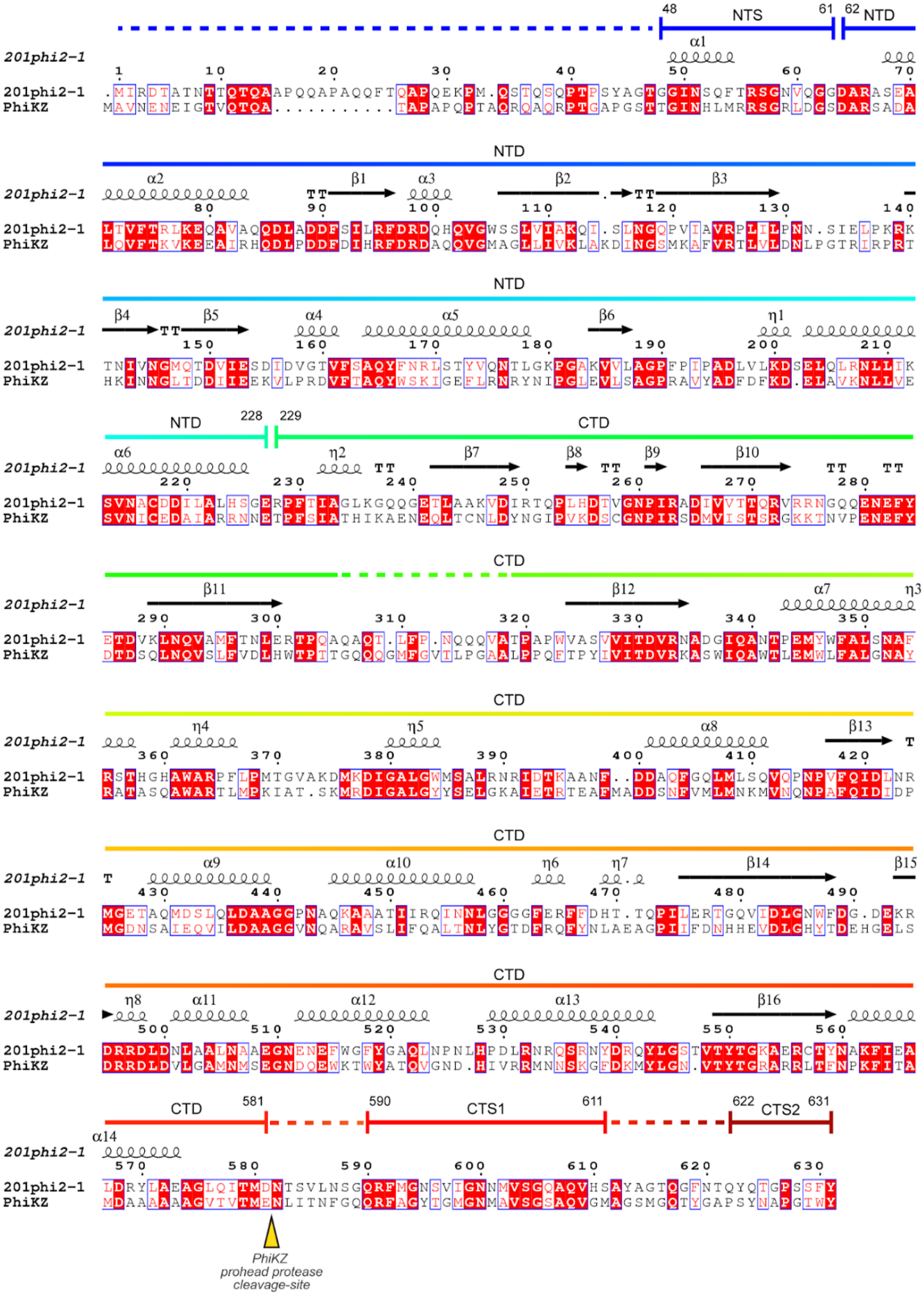

Supplementary Figure 1. Alignment of 201phi2-1 and phiKZ chimallin proteins.

SI Table 1 | *In situ* cryoFIB-ET data collection and reconstruction statistics

	201phi2-1 infected *P. chlororaphis*					Goslar infected *E. coli* (APEC2248)				
		chimallin						chimallin		
	70S (EMD-25183)	Consensus (EMD-25221)	Concave (EMD-25220)	Flat (EMD-25222)	Convex (EMD-25223)	50S (EMD-25360)	70S (EMD-25359)	Consensus (EMD-25229)	Concave (EMD-25262)	Convex (EMD-25358)
**Data collection**										
Magnification			33,000					42,000		
Voltage (kV)	300									
Defocus range (µm)			-4.5 to -6					-5 to -6		
Pixel size (Å)			3.46					4.27		
Electron exposure (e–/Å^2)			120					150		
Tilt-range/step (°)			±51 / 3					±56 / 2		
Tilt-scheme	dose-symmetric, grouping 2									
**Processing**										
Symmetry imposed	C1	C4	C4	C4	C4	C1	C1	C4	C4	C4
Initial particle images (no.)	17,169	66,887		8,454		98,981		42,416	4,501	
Final particle images (no.)	11,148	8,454	2,033	4,475	945	3,710	46,056	4,501	2,802	1,699
Map resolution (Å)	10	24	20	18	23	12	8.5	27	20	25
FSC threshold	0.143									

SI Table 2 | Cryo-EM data collection, reconstruction, and refinement statistics

		201phi2-1 gp105			Goslar gp189	
	24mer (EMD-25390) (PDB 7SQQ)	tetramer (EMD-25391) (PDB 7SQR)	monomer (EMD-25392) (PDB 7SQS)	24mer (EMD-25394) (PDB 7SQT)	tetramer (EMD-25395) (PDB 7SQU)	monomer (EMD-25396) (PDB 7SQV)
**Data collection and processing**						
Magnification		130,000			165,000	
Voltage (kV)			300			
Electron exposure (e–/Å^2)		43			40	
Defocus range (μm)			-0.1 to -1.5			
Pixel size (Å)		1.075			0.8452	
Symmetry imposed	O	C4	C1	O	C4	C1
Initial particle images (no.)	140,782	772,788	2,657,452	289,387	471,192	1,407,340
Final particle images (no.)	128,798	664,363	2,657,452	78,532	351,835	1,407,340
Map resolution (Å)	4.2	3.4	3.1	4.0	2.6	2.3
FSC threshold			0.143			
Map resolution range (Å)	4.0 to 5.1	3.3 to 4.4	2.9 to 4.8	3.7 to 6.0	2.5 to 3.4	2.3 to 3.4
**Refinement**						
Initial model used (PDB code)		*de novo*			7SQS	
Model resolution (Å)	4.7	3.6	3.3	4.4	2.8	2.5
FSC threshold			0.5			
Model composition						
Non-hydrogen atoms	103,632	17,272	4,376	107,136	17,856	4,471
Protein residues	13,296	2,216	560	13,752	2,292	573
Ligands	0	0	0	0	0	7
B factors (Å^2)						
Protein	408.17	125.69	108.00	318.81	97.77	77.87
Ligand (waters)	n.a.	n.a.	n.a.	n.a.	n.a.	71.61
R.m.s. deviations						
Bond lengths (Å)	0.003	0.003	0.002	0.010	0.010	0.001
Bond angles (°)	0.633	0.632	0.469	0.699	0.698	0.380

Validation						
MolProbity score	1.63	1.56	1.31	1.16	0.94	0.87
Clashscore	8.23	6.82	3.59	2.13	1.16	1.36
Poor rotamers (%)	0	0	0.22	1.25	1.04	0.63
Ramachandran plot						
Favored (%)	96.89	96.89	97.08	97.53	97.53	99.29
Allowed (%)	3.11	3.11	2.92	2.47	2.47	0.71
Disallowed (%)	0	0	0	0	0	0

SI Table 3 | Summary of *in vitro* 201phi2-1 chimallin protomer interfaces

Chain-1	Atoms	Residues	Chain-2	Atoms	Residues	Area (Å^2)	H bonds	Salt bridge	Cation-π
A	28	9	F	28	9	307	0	0	0
A	130	34	H	147	43	1253	7	6	0
A	147	43	B	130	34	1253	7	6	0
A	198	53	D	205	56	1945	14	0	1
A	205	56	G	198	53	1945	14	0	1

SI Table 4 | Summary of a 201phi2-1 chimallin protomer's polar interactions *in vitro*

Chain-1		Resi	Resn	Atom	Chain-2		Resi	Resn	Atom	Dist (Å)	Type
A	B	48	GLY	N	H	A	287	ASP	OD2	3.6	H bond
A	B	51	ASN	NE2	H	A	337	GLY	O	3.2	H bond
A	B	51	ASN	OD1	H	A	339	GLN	N	3.4	H bond
A	B	56	ARG	O	H	A	339	GLN	NE2	2.6	H bond
A	B	59	ASN	N	H	A	86	ASP	O	3.5	H bond
A	B	102	GLN	OE1	H	A	281	ASN	ND2	2.3	H bond
A	B	163	SER	OG	H	A	282	GLU	OE2	3.1	H bond
A	B	56	ARG	NE	H	A	219	ASP	OD1	2.5	Salt bridge
A	B	56	ARG	NH1	H	A	227	GLU	OE1	3.4	Salt bridge
A	B	56	ARG	NH1	H	A	227	GLU	OE2	3.7	Salt bridge
A	B	56	ARG	NH2	H	A	227	GLU	OE1	3.9	Salt bridge
A	B	56	ARG	NH2	H	A	227	GLU	OE2	2.9	Salt bridge
A	B	56	ARG	NH2	H	A	219	ASP	OD1	3.3	Salt bridge
A	G	319	THR	OG1	D	A	319	THR	N	2.9	H bond
A	G	406	GLN	OE1	D	A	306	GLN	N	3.9	H bond
A	G	424	ARG	NH2	D	A	624	GLN	O	3.3	H bond
A	G	430	GLN	OE1	D	A	629	SER	N	3.8	H bond
A	G	430	GLN	OE1	D	A	629	SER	OG	2.8	H bond
A	G	437	ASP	OD2	D	A	631	TYR	OH	2.8	H bond
A	G	592	PHE	O	D	A	516	TRP	NE1	2.3	H bond
A	G	595	ASN	N	D	A	301	ARG	O	3.5	H bond
A	G	604	SER	N	D	A	565	GLU	OE2	3	H bond
A	G	604	SER	OG	D	A	565	GLU	OE2	3.4	H bond
A	G	605	GLY	N	D	A	565	GLU	OE2	3.6	H bond
A	G	606	GLN	NE2	D	A	358	HIS	O	3.1	H bond
A	G	607	ALA	N	D	A	357	THR	OG1	3.8	H bond

A	G	610	HIS	N	D	A	578	ILE	O	3.1	H bond
A	G	446	LYS	NZ	D	A	631	TYR	-	5.8	Cation-π

SI Table 5 | Gaussian network model hinge residues for 201phi2-1 chimallin

resi	resn	Mode 1	Mode 2	Mode 3	Mode 4	Mode 5	total
45	ALA	1	2	2	4	4	13
46	GLY	5	5	4	-	-	14
47	THR	-	1	1	-	2	4
50	ILE	-	-	1	-	-	1
51	ASN	1	-	-	-	-	1
60	VAL	-	-	1	-	-	1
62	GLY	2	3	2	-	-	7
63	GLY	4	3	3	-	-	10
64	ASP	-	-	1	-	-	1
66	ARG	-	-	1	-	-	1
97	ASP	-	-	1	-	-	1
106	SER	-	-	1	-	-	1
132	ASN	-	-	1	-	-	1
138	LYS	-	-	1	-	-	1
161	VAL	-	-	1	-	-	1
163	SER	-	-	1	-	-	1
166	TYR	-	-	1	-	-	1
239	GLN	-	-	1	-	-	1
241	GLU	-	-	1	-	-	1
242	THR	-	-	1	-	-	1
243	LEU	-	-	1	-	-	1
244	ALA	-	-	1	-	-	1
271	GLN	-	-	1	-	-	1
276	ASN	1	1	1	-	-	3
277	GLY	3	2	2	-	-	7
278	GLN	1	2	1	-	-	4
279	GLN	1	1	1	-	-	3
280	GLU	-	-	1	-	-	1
284	TYR	-	1	1	-	-	2
285	GLU	3	2	2	-	-	7
286	THR	3	3	3	-	-	9
289	LYS	-	-	1	-	-	1
336	ASP	-	-	1	-	-	1
337	GLY	-	-	1	-	-	1
358	HIS	-	-	1	-	-	1
368	PRO	-	-	1	-	-	1
370	THR	2	1	2	-	-	5
371	GLY	1	1		-	-	2
372	VAL	3	2	2	-	-	7
373	ALA	-	1	1	-	-	2
375	ASP	-	-	1	-	-	1
396	ALA	-	-	1	-	-	1
397	ALA	-	-	1	-	-	1
400	ASP	-	-	1	-	-	1
441	GLY	-	-	-	-	1	1
442	PRO	-	-	-	-	1	1
446	LYS	-	-	1	-	-	1
526	ASN	-	-	1	-	-	1
538	ASN	-	-	1	-	-	1
541	ARG	-	-	1	-	-	1
543	TYR	-	-	1	-	-	1
545	GLY	-	-	1	-	-	1
546	SER	-	-	1	-	-	1
568	ASP	-	-	1	-	-	1
580	MET	-	-	1	-	-	1
582	ASN	-	-	-	-	1	1
583	THR	-	-	-	4	1	5
584	SER	1	2	1	3	5	12

585	VAL	-	-	1	-		1
586	LEU	-	-	-	5	5	10
587	ASN	-	-	-	-	1	1
588	SER	1	1	2	-	-	4
589	GLY	-	-	1	-	-	1
596	SER	-	1	-	1	-	2
606	GLN	-	-	1	-	-	1
607	ALA	1	-	1	1	-	3
611	SER	-	-	1	-	-	1
612	ALA	4	1	6	4	10	25
	**total**	38	36	78	22	311	

SI Table 6 | Pore-facing residues during 201phi2-1 chimallin simulations

Center 4-fold

resi	resn	count
69	GLU	2
72	THR	6
76	ARG	9
79	GLU	9
80	GLN	6
83	ALA	5
131	ASN	1
133	SER	1
196	ASP	2
198	VAL	9
200	LYS	9
201	ASP	8
203	ARG	1
203	GLU	3
372	VAL	4
373	ALA	1
374	LYS	8
375	ASP	2
377	LYS	1
378	ASP	1
391	ARG	5
395	LYS	4
480	GLY	1
488	PHE	9
490	GLY	8
491	ASP	4
493	LYS	6
509	ASP	1
509	GLU	8
511	ASN	1
546	SER	5
547	THR	5

Corner 4-fold

resi	resn	count
117	ASN	3
119	GLN	2
303	PRO	1
304	GLN	1
305	ALA	1
307	ALA	1
309	THR	1
311	PHE	1
312	PRO	1
317	VAL	1
319	THR	1
320	PRO	2
414	PRO	1
415	ASN	1
445	GLN	1
452	ILE	1
453	ARG	2
456	ASN	1
464	GLU	4
465	ARG	2
466	PHE	1
468	ASP	3
469	HIS	1
470	THR	4
471	THR	4
472	GLN	2
562	LYS	2
569	ARG	1
573	GLU	1
596	SER	1
600	ASN	3
601	ASN	1
602	MET	1
604	SER	2
606	GLN	1
607	ALA	1

SI Table 7 | Bacteriophage chimallin proteins

**Genbank ID**	**Protein ID**	**Scientific Name**
QDB70465.1	gp052	*Aeromonas* phage CF8
QDJ97041.1	gp044	*Aeromonas* phage D3
QDH47031.1	gp211	*Aeromonas* phage LAh10
QDJ96807.1	gp048	*Aeromonas* phage PS1
BCM95275.1	gp239	*Burkholderia* phage FLC6
YP_008125763.1	gp023	*Cronobacter* phage CR5
YP_009821902.1	gp002	*Edwardsiella* phage pEt-SU
YP_009005014.1	gp219	*Erwinia* phage Ea35-70
QQO90522.1	gp038	*Erwinia* phage pEa_SNUABM_43
YP_009010075.1	gp022	*Erwinia* phage PhiEaH1
YP_007237854.1	gp204	*Erwinia* phage phiEaH2
YP_009290657.1	gp039	*Erwinia* phage vB_EamM_Asesino
YP_009292105.1	gp248	*Erwinia* phage vB_EamM_Caitlin
YP_009292727.1	gp253	*Erwinia* phage vB_EamM_ChrisDB
YP_009278336.1	gp024	*Erwinia* phage vB_EamM_EarlPhillipIV
YP_009293007.1	gp244	*Erwinia* phage vB_EamM_Huxley
YP_009278639.1	gp265	*Erwinia* phage vB_EamM_Kwan
YP_009283513.1	gp024	*Erwinia* phage vB_EamM_Phobos
ANH52002.1	gp222	*Erwinia* phage vB_EamM_RAY
YP_009612772.1	gp028	*Erwinia* phage vB_EamM_RisingSun
YP_009606010.1	gp299	*Erwinia* phage vB_EamM_Simmy50
QKN85862.1	gp240	*Escherichia* phage vB_EcoM_EC001
*** YP_009820873.1**	**gp189**	***Escherichia* phage vB_EcoM_Goslar**
YP_006383413.1	gp133	*Halocynthia* phage JM-2012 (infects *Vibrio*)
QPB09376.1	gp281	*Klebsiella* phage Miami
QGH71899.1	gp036	*Klebsiella* phage N1M2
QQV92022.1	gp083	*Klebsiella* phage vB_KpM_FBKp24

CAD5236077.1	gp088	*Klebsiella* phage vB_KvM-Eowyn
YP_009853356.1	gp004	*Photobacterium* phage PDCC-1
QMP24145.1		*Proteus* phage 10
QNN97444.1		*Proteus* phage 7
*** YP_001956829.1**	**gp105**	***Pseudomonas* phage 201phi2-1**
* YP_418056.1	gp023	*Pseudomonas* phage EL
ANM44831.1	gp073	*Pseudomonas* phage KTN4
YP_009608965.1	gp050	*Pseudomonas* phage Noxifer
YP_004957964.1	gp057	*Pseudomonas* phage OBP
BBI55859.1	gp186	*Pseudomonas* phage PA02
QBX32206.1		*Pseudomonas* phage PA1C
YP_009617618.1	gp330	*Pseudomonas* phage PA7
ARV76713.1	gp082	*Pseudomonas* phage Phabio
* YP_009217136.1	gp054	*Pseudomonas* phage phiPA3
QBJ02586.1	gp056	*Pseudomonas* phage Psa21
YP_009619844.1	gp304	*Pseudomonas* phage SL2
QOV07924.1		*Pseudomonas* phage vB_PaeM_kmuB
QEM41783.1		*Pseudomonas* phage vB_PaeM_PS119XW
* NP_803620.1	gp054	*Pseudomonas* virus phiKZ
YP_009598981.1	gp133	*Ralstonia* phage RP12
* BAW19549.1	gp286	*Ralstonia* phage RP31
YP_009208033.2	gp229	*Ralstonia* phage RSF1
* YP_009213072.1	gp223	*Ralstonia* phage RSL2
QNI20738.1		*Salmonella* phage JN03
QOC54489.1	gp029	*Salmonella* phage pSal-SNUABM-04
* YP_009153316.1	gp244	*Salmonella* phage SPN3US
AWN08967.1	gp006	*Salmonella* phage STsAS
QKE54546.1	gp230	*Salmonella* phage vB_SalM_SA002
YP_009833990.1	gp310/311/312	*Serratia* phage 2050HW
YP_009849136.1	gp040	*Serratia* phage Moabite

* QFP93061.1		*Serratia* phage PCH45
YP_009843316.1	gp214	*Vibrio* phage 2 TSL-2019
* YP_009622192.1	gp065	*Vibrio* phage Aphrodite1
* AXH70812.1	gp071	*Vibrio* phage BONAISHI
YP_009599417.1	gp130	*Vibrio* phage pTD1
AQT28149.1	gp208	*Vibrio* phage pVa-21
YP_009847864.1	gp128	*Vibrio* phage USC-1
QNI21050.1		*Vibrio* phage vB_pir03
QAX96061.1		*Vibrio* phage vB_VmeM-Yong XC31
YP_009626053.1	gp077	*Vibrio* phage VP4B
QNR51901.1	gp210	*Xanthomonas* phage Xoo-sp14

SI Table 8 | Summary of *in vitro* Goslar chimallin protomer interfaces

Chain-1	Atoms	Residues	Chain-2	Atoms	Residues	Area (Å^2)	H bonds	Salt bridge	Cation-π
A	11	3	F	11	3	91	0	2	0
A	164	38	H	210	62	1779	18	11	1
A	210	62	B	164	38	1779	18	11	1
A	206	54	D	210	58	2024	16	6	3
A	210	58	G	206	56	2024	16	6	3

SI Table 9 | Summary of a Goslar chimallin protomer’s polar interactions *in vitro*

Chain-1		Resi	Resn	Atom	Chain-2		Resi	Resn	Atom	Dist (Å)	Type
A	F	140	HIS	NE2	F	A	427	ASP	OD2	3.2	Salt bridge
A	B	46	MET	O	H	A	279	GLN	NE2	3.5	H bond
A	B	50	ASN	ND2	H	A	330	GLU	O	3.4	H bond
A	B	50	ASN	OD1	H	A	332	ASP	N	3.5	H bond
A	B	55	ARG	NE	H	A	206	ASN	OD1	3.1	H bond
A	B	55	ARG	NH2	H	A	210	THR	OG1	3.2	H bond
A	B	56	ARG	NH1	H	A	107	SER	OG	3.7	H bond
A	B	56	ARG	NH2	H	A	209	GLN	OE1	3.4	H bond
A	B	56	ARG	NH2	H	A	107	SER	OG	3.6	H bond
A	B	57	ILE	N	H	A	205	ASP	OD2	3.1	H bond
A	B	57	ILE	O	H	A	202	SER	OG	3.6	H bond
A	B	59	ARG	NH1	H	A	332	ASP	O	3.7	H bond
A	B	59	ARG	NH2	H	A	331	LEU	O	3.1	H bond
A	B	60	ASN	ND1	H	A	329	LEU	O	3.3	H bond
A	B	97	SER	O	H	A	275	TYR	OH	3.2	H bond
A	B	130	PRO	O	H	A	278	GLN	NE2	2.7	H bond
A	B	152	ASN	ND2	H	A	276	ASP	O	3.3	H bond
A	B	152	ASN	OD1	H	A	276	ASP	N	3.3	H bond
A	B	154	ASN	ND2	H	A	273	SER	O	3.3	H bond
A	B	55	ARG	NH1	H	A	213	GLU	OE1	3.8	Salt bridge
A	B	55	ARG	NH1	H	A	213	GLU	OE2	3.1	Salt bridge
A	B	55	ARG	NH2	H	A	213	GLU	OE1	3.4	Salt bridge
A	B	56	ARG	NE	H	A	205	ASP	OD1	3.1	Salt bridge
A	B	56	ARG	NH1	H	A	85	ASP	OD2	3.3	Salt bridge
A	B	56	ARG	NH2	H	A	205	ASP	OD1	3.1	Salt bridge
A	B	134	ARG	NE	H	A	276	ASP	OD2	3.9	Salt bridge
A	B	134	ARG	NH2	H	A	274	ASP	OD1	3.5	Salt bridge

A	B	134	ARG	NH2	H	A	274	ASP	OD2	3.2	Salt bridge
A	B	134	ARG	NH2	H	A	276	ASP	OD2	3.6	Salt bridge
A	B	147	ARG	NH1	H	A	276	ASP	OD2	3.6	Salt bridge
A	B	56	ARG	CZ	H	A	86	PHE	-	5.6	Cation-π
A	G	399	TYR	OH	D	A	300	ASN	N	3.5	H bond
A	G	403	GLN	NE2	D	A	297	PRO	O	3.4	H bond
A	G	427	ARG	NH2	D	A	628	PHE	O	2.5	H bond
A	G	593	ARG	NH2	D	A	517	GLU	O	4.7	H bond
A	G	595	ASN	N	D	A	292	TYR	O	2.9	H bond
A	G	595	ASN	O	D	A	293	SER	OG	2.9	H bond
A	G	599	ARG	NE	D	A	293	SER	OG	3.6	H bond
A	G	599	ARG	NH2	D	A	294	PRO	O	3.3	H bond
A	G	599	ARG	NH2	D	A	293	SER	OG	3.7	H bond
A	G	603	LEU	N	D	A	410	LEU	O	2.7	H bond
A	G	603	LEU	O	D	A	410	LEU	N	3.6	H bond
A	G	604	GLY	O	D	A	351	ASN	ND2	3.5	H bond
A	G	605	ASN	O	D	A	566	GLN	NE2	2.8	H bond
A	G	610	ILE	O	D	A	579	ILE	N	2.9	H bond
A	G	611	TYR	OH	D	A	348	ALA	O	3.2	H bond
A	G	612	ALA	N	D	A	579	ILE	O	3.4	H bond
A	G	593	ARG	NE	D	A	521	ASP	OD1	3.0	Salt bridge
A	G	593	ARG	NH1	D	A	520	GLU	OE1	3.5	Salt bridge
A	G	593	ARG	NH2	D	A	521	ASP	OD1	3.6	Salt bridge
A	G	593	ARG	NH2	D	A	520	GLU	OE1	2.6	Salt bridge
A	G	601	ARG	NH1	D	A	559	ASP	OD1	3.4	Salt bridge
A	G	601	ARG	NH1	D	A	559	ASP	OD2	3.1	Salt bridge
A	G	427	ARG	CZ	D	A	631	PHE	-	4.3	Cation-π
A	G	593	ARG	CZ	D	A	292	TYR	-	5.2	Cation-π
A	G	599	ARG	CZ	D	A	407	TYR	-	5.8	Cation-π

SI Table 10 | Bacterial strains and plasmids used in this study

**Bacterial strain**	**Source**
Pseudomonas chlororaphis 200-B	[88]
Escherichia coli APEC 2248	DSMZ (dsmz.de) #103255
Escherichia coli Rosetta2 pLysS	EMD Millipore

**Plasmid**	**Host strain**	**Source**
pHERD30-T	*P. chlororaphis*	[89]
pHERD30-T + GFPmut1	*P. chlororaphis*	[90]
pHERD30-T + 201phi2-1 GFP-chimallin full-length	*P. chlororaphis*	this study
pHERD30-T + 201phi2-1 GFP-chimallin 48-631	*P. chlororaphis*	this study
pHERD30-T + 201phi2-1 GFP-chimallin 64-631	*P. chlororaphis*	this study
pHERD30-T + 201phi2-1 GFP-chimallin 1-611	*P. chlororaphis*	this study
pHERD30-T + 201phi2-1 GFP-chimallin 1-581	*P. chlororaphis*	this study
pHERD30-T + 201phi2-1 GFP-chimallin 48-611	*P. chlororaphis*	this study
pHERD30-T + 201phi2-1 GFP-chimallin 64-581	*P. chlororaphis*	this study
2-BT (His6-TEV site fusion expression vector)	*E. coli* Rosetta2 pLysS	Addgene (addgene.org) #29666
2-BT + 201phi2-1 chimallin FL	*E. coli* Rosetta2 pLysS	this study
2-BT + 201phi2-1 chimallin 48-631	*E. coli* Rosetta2 pLysS	this study

2-BT + 201phi2-1 chimallin 64-631	*E. coli* Rosetta2 pLysS	this study
2-BT + 201phi2-1 chimallin 1-612	*E. coli* Rosetta2 pLysS	this study
2-BT + 201phi2-1 chimallin 1-582	*E. coli* Rosetta2 pLysS	this study
2-BT + 201phi2-1 chimallin 48-612	*E. coli* Rosetta2 pLysS	this study
2-BT + 201phi2-1 chimallin 64-582	*E. coli* Rosetta2 pLysS	this study
2-BT + Goslar chimallin full-length	*E. coli* Rosetta2 pLysS	this study
