# Supplementary material for: Architecture and self-assembly of the jumbo bacteriophage nuclear shell
Source: Nature. 2022 Aug 3;608(7922):429–35. doi: 10.1038/s41586-022-05013-4 (PMC9365700; doi:10.1038/s41586-022-05013-4)
Supplement: Supplementary file 2 — Reporting Summary [file 41586_2022_5013_MOESM2_ESM.pdf]

nature portfolio

Corresponding author(s): Joe Pogliano, Kevin D. Corbett, Elizabeth Villa

Last updated by author(s): May 4, 2022

Reporting Summary

Nature Portfolio wishes to improve the reproducibility of the work that we publish. This form provides structure for consistency and transparency in reporting. For further information on Nature Portfolio policies, see our Editorial Policies and the Editorial Policy Checklist.

Statistics

For all statistical analyses, confirm that the following items are present in the figure legend, table legend, main text, or Methods section.

n/a	Confirmed	
☐	☒	The exact sample size (*n*) for each experimental group/condition, given as a discrete number and unit of measurement
☐	☒	A statement on whether measurements were taken from distinct samples or whether the same sample was measured repeatedly
☐	☒	The statistical test(s) used AND whether they are one- or two-sided<br>*Only common tests should be described solely by name; describe more complex techniques in the Methods section.*
☒	☐	A description of all covariates tested
☐	☒	A description of any assumptions or corrections, such as tests of normality and adjustment for multiple comparisons
☐	☒	A full description of the statistical parameters including central tendency (e.g. means) or other basic estimates (e.g. regression coefficient) AND variation (e.g. standard deviation) or associated estimates of uncertainty (e.g. confidence intervals)
☐	☒	For null hypothesis testing, the test statistic (e.g. *F*, *t*, *r*) with confidence intervals, effect sizes, degrees of freedom and *P* value noted<br>*Give P values as exact values whenever suitable.*
☒	☐	For Bayesian analysis, information on the choice of priors and Markov chain Monte Carlo settings
☒	☐	For hierarchical and complex designs, identification of the appropriate level for tests and full reporting of outcomes
☒	☐	Estimates of effect sizes (e.g. Cohen's *d*, Pearson's *r*), indicating how they were calculated

Our web collection on statistics for biologists contains articles on many of the points above.

Software and code

Policy information about availability of computer code

Data collection	DeltaVision softWoRx program-v6.5.2, SerialEM-v3.8b11, ASTRA v.8
Data analysis	DeltaVision softWoRx program-v6.5.2, FIJI-v2.1.0/1.53c, MATLAB-2019a, IMOD-v4.10.28, Warp/M-v1.09, Cube-commit-aa59444, RELION-v3.1.1, Dynamo-v1.1.514,PlaceObjects-1.0.0, UCSF-Chimera-v1.15, dynamo2m-v0.2.2, TomoSegMemTV-vApr2020, Amira-v6.7, PyCurv-commit-fa70ce7, ASTRA v. 8 software, Prism-v9.3, cryoSPARC-v3.2, 3DFSC-webserver-v3, DeepTracer-webserver-v1, COOT-v0.9.1, PHENIX-v1.19.2, MolProbity-v4.5.1, EMRinger-v1.0.0, ePISA-v1.5254 web server, CaPTURE-webserver-v1, DaReUS-Loop-v1, APBS-v3.0.0, PROPKA-v3.4.0, PDB2PQR-v3.4.0, ProDy-v1.0, Amber 19ffsb (Amber 20), CPPTRAJ-v.25.6, MDTraj-v1.9.4, CHAP-v0.9.1, PyMOL-v2.5, ChimeraX-v1.2.5, VMD-1.9.4a35, SHAKE (Amber 19ffsb), Particle mesh Ewald (Amber 19ffsb), Langevin thermostat (Amber 19ffsb), Berendsen barostat (Amber 19ffsb).

For manuscripts utilizing custom algorithms or software that are central to the research but not yet described in published literature, software must be made available to editors and reviewers. We strongly encourage code deposition in a community repository (e.g. GitHub). See the Nature Portfolio guidelines for submitting code & software for further information.

Data

Policy information about availability of data

All manuscripts must include a data availability statement. This statement should provide the following information, where applicable:

- Accession codes, unique identifiers, or web links for publicly available datasets
- A description of any restrictions on data availability
- For clinical datasets or third party data, please ensure that the statement adheres to our policy

Cryo-EM density maps have been deposited in the Electron Microscopy Data Bank. Subtomogram averaging maps have the accession numbers: EMD-25221

(201phi2-1, consensus), EMD-25220 (201phi2-1, concave), EMD-25222 (201phi2-1, flat), EMD-25223 (201phi2-1, convex), EMD-25183 (P. chlororaphis, 70S), EMD-25229 (Goslar, consensus), EMD-25262 (Goslar, concave), EMD-25358 (Goslar, convex), EMD-25359 (APEC2248, 70S), EMD-25360 (APEC2248, 50S). Single-particle maps have the accession numbers: EMD-25393 (201phi2-1, O), EMD-25391 (201phi2-1, C4), EMD-25392 (201phi2-1, C1), EMD-25393 (201phi2-1, D4) EMD-25394 (Goslar, O), EMD-25395 (Goslar, C4), and EMD-25395 (Goslar, C1). Coordinate models have been deposited in the RCSB Protein Data Bank with the accession numbers 7SQQ (201phi2-1, O), 7SQR (201phi2-1, C4), 7SQS (201phi2-1 C1 monomer), 7SQT (Goslar, O), 7SQU (Goslar, C4), and 7SQV (Goslar, C1). Raw cryo-EM data have been deposited with the Electron Microscopy Public Image Archive with accession codes: EMPIAR-10859 (in situ 201phi2-1 tilt-series), EMPIAR-10860 (in situ Goslar tilt-series), EMPIAR-10862 (in vitro 201phi2-1 frame-series), and EMPIAR-10863 (in vitro Goslar frame-series). Genbank IDs for protein sequences used in this study are provided in SI Table 7. All other data are available upon request to the corresponding author(s).

Field-specific reporting

Please select the one below that is the best fit for your research. If you are not sure, read the appropriate sections before making your selection.

☒ Life sciences ☐ Behavioural & social sciences ☐ Ecological, evolutionary & environmental sciences

For a reference copy of the document with all sections, see nature.com/documents/nr-reporting-summary-flat.pdf

Life sciences study design

All studies must disclose on these points even when the disclosure is negative.

Sample size	Sample sizes for were not predetermined and were set by instrument and computational resource availability, as well as the area available for imaging per session in the case of microscopy experiments.
Data exclusions	1) Poorly aligned tilt-series and micrographs were excluded based on CTF-fit quality, as these data are known to negatively impact the quality of subsequent averaging and analysis procedures. 2) The intertetramer ("corner four-fold") pore in the upper-left quadrant contained two frames that caused CHAP to crash; these frames were removed before averaging after consultation with the CHAP developers. Considering we still averaged 1502 frames x 4 pores - 2 bad frames = 6006 frames for the intertetramer pores, we do not feel that this removal causes any difference in our conclusions.
Replication	Cryo-FIB/ET of infections was performed independently twice with reproducible results. Cryo-EM samples were technically replicated four times. Fluorescence microscopy and growth curve experiments were performed independently twice for each sample with at least two technical replicates in each instance (specific values indicated in manuscript). SEC-MALS were replicated from independent sample preparations.Molecular dynamics simulations were initialized for five runs and resulted in similar, reproducible trajectories.
Randomization	Half-sets for the single-particle cryo-EM data were assigned randomly automatically by RELION-v3.1.1 during the first 3D-auto refinement run. Half-sets for the subtomogram analysis were composed on a per-surface basis in order to achieve roughly even sets. This non-random assignment is necessary as the close-packing of the lattice leads to adjacent subtomograms containing some overlapping or identical information and noise. Thus, splitting adjacent subtomograms randomly into half-sets has a high chance of invalidating assumptions of the method used for resolution estimation, which assumes noise is independent between the half-sets.
Blinding	Blinding is not relevant to this study. Researchers were not blinded to the identity of the samples as it was not technically or practically feasible to do so. For cryo-EM experiments at the resolutions achieved in this study, prior knowledge of the sample is essential for reliable interpretation of density features.

Reporting for specific materials, systems and methods

We require information from authors about some types of materials, experimental systems and methods used in many studies. Here, indicate whether each material, system or method listed is relevant to your study. If you are not sure if a list item applies to your research, read the appropriate section before selecting a response.

Materials & experimental systems

n/a	Involved in the study	
☒	☐	Antibodies
☒	☐	Eukaryotic cell lines
☒	☐	Palaeontology and archaeology
☒	☐	Animals and other organisms
☒	☐	Human research participants
☒	☐	Clinical data
☒	☐	Dual use research of concern

Methods

n/a	Involved in the study	
☒	☐	ChIP-seq
☒	☐	Flow cytometry
☒	☐	MRI-based neuroimaging
